# Single-Pixel Imaging with Origami Pattern Construction

**DOI:** 10.3390/s19235135

**Published:** 2019-11-23

**Authors:** Wen-Kai Yu, Yi-Ming Liu

**Affiliations:** 1 Center for Quantum Technology Research, School of Physics, Beijing Institute of Technology, Beijing 100081, China; 2 Key Laboratory of Advanced Optoelectronic Quantum Architecture and Measurements of Ministry of Education, School of Physics, Beijing Institute of Technology, Beijing 100081, China; 3 Department of Physics, Beijing Normal University, Beijing 100875, China

**Keywords:** single-pixel imaging, computational imaging, compressed sensing, ghost imaging, pattern construction, efficient data acquisition, sub-Nyquist sampling, image reconstruction

## Abstract

Single-pixel compressive imaging can recover images from fewer measurements, offering many benefits especially for the imaging modalities where array detection is unavailable. However, the widely used random projections fail to explore internal relations between coding patterns and image reconstruction. Here, we propose a single-pixel imaging method based on a deterministic origami pattern construction that can lead to a more accurate pattern ordering sequence and better imaging quality. It can decrease the sampling ratio, closer to the upper bounds. The experimental realization of this approach is a big step forward towards practical applications.

## 1. Introduction

Imaging is extremely important for acquiring the light field information of the target [1]. Single-pixel imaging (SPI) [2,3], as a novel imaging alternative, can obtain images by using only a point detector without spatial resolution, having extraordinary application prospects, especially at non-visible wavelengths. Except for point-by-point scanning, SPI has also developed some imaging techniques based on Fourier [4] or Hadamard transforms [5,6,7] (i.e., complete orthogonal basis modulation, full sampling). Both Fourier and Hadamard SPI are computational imaging techniques. It is worth mentioning that there is another computational imaging technology named ghost imaging (GI) [8], which acquires the object image by correlating the intensity signals recorded in both reference arm and object arm. GI generally requires random modulation and oversampling, it has several modalities, such as quantum GI [8], thermal light GI [9], pseudo-thermal light GI [10] and computational GI [11,12,13]. In the first three schemes, the reference patterns usually need to be captured by a scanning detector or a spatially resolved array imager. While in computational GI, the reference patterns can be precomputed from a programmable spatial light modulator, thus the imaging apparatus can be simplified to one optical beam without reference arm and one can compute the correlation between single-pixel intensities and the known patterns. Therefore, among various GI schemes, only computational GI can be regarded as SPI. Lately, with the rapid development of information theory, the single-pixel camera [14] based on compressed sensing (CS) [15,16] has been implemented. This technology can obtain images of sparse/compressive targets at very low sampling (subsampling) rates, breaking the Nyquist-Shannon sampling limit. Generally, there is a trade-off between sparsity and subsampling, and the optimal recovery order of *M* (the measurement number) proportional to *k* (the object sparsity) can be cast in the phase transition framework of CS [17,18]. To our knowledge, the phase transition is bounded by two alternative methods: the polytope analysis [19] and the geometric functional analysis [20]. To some extent, single-pixel compressive imaging shares the same mathematical measurement model with thermal light, pseudo-thermal light or computational GI, all of y=Ax+e, where *y* denotes the single-pixel/bucket measured vector, *A* is a measurement matrix consisting of *M* rows, each of which is stretched from every modulated/reference pattern, and *e* stands for the measurement noise. But their reconstruction principles are different, the former relies upon sparse recovery, and the latter applies correlation functions. Generally, the computational complexity of sparse recovery is much higher than that of correlation functions. For common resolutions, one can obtain good performance via random compressive sampling when the sampling ratio is higher than 30% (an empirical value, because the sparsity of natural images cannot be very low) [21], which has become the bottleneck of compressive imaging for realizing real-time practical applications, especially in large pixel-scale imaging.

Recent studies have demonstrated that not all patterns contribute to the reconstructions of computational imaging, resulting in a technology named correspondence imaging (CI) [22,23,24,25,26], where the reference patterns with the corresponding single-pixel/bucket values well above or below the mean of detected values are picked up for reconstruction after oversampling the object image. Since there is such a phenomenon, why not just construct a universal deterministic (instead of random) measurement matrix where the modulated patterns with the biggest contributions to the reconstruction are preferentially displayed. In particular, an earlier research work [21] reported a GI method based on “Russian Dolls” (RD) ordering of the Hadamard basis to improve imaging efficiency, which provided us with inspiration.

In this paper, we propose an origami model to construct deterministic patterns for SPI. The row vectors stretched from each constructed pattern happen to be orthogonal to each other. It turns out that the origami patterns are reordered Hadamard patterns. Under the CS framework, the sampling ratio required by this method can approach the bounds limited by phase transition. This technique may open a door to practical compressive video applications [27,28] with fewer measurements.

## 2. Theory and Methods

Before we start introducing our approach, we first need to present some GI principles and related consensus. Given the consistency of the measurement models of GI and CS, some conclusions found in GI can be easily extended to CS. In GI fields, according to the theory of CI [22,23,24,25,26], a positive (or negative) ghost image can be retrieved by only averaging some small fractions of the reference subset $I_{R}^{+}$ (or $I_{R}^{-}$), i.e., $\langle I_{R_{+}}\rangle$ (or $\langle I_{R_{-}}\rangle$), corresponding to $\left\{S_{B}^{+}|S_{B}\gg \langle S_{B}\rangle \right\}$ (or $\left\{S_{B}^{-}|S_{B}\ll \langle S_{B}\rangle \right\}$). Although only a small number of patterns are involved in the calculation, the total number of measurements is not reduced, and the conditional averaging of reference patterns is made after oversampling. The mechanism behind it was firstly elucidated with some attempts [24,25], and recently was strictly explained through a probability theory, which regards the light intensities as stochastic variables and uses a joint probability density function, followed by an analysis of the visibility and contrast-to-noise ratio (reconstruction quality) vs. the threshold parameter (i.e., former percentage) and the pixel number of the object part [29]. According to this theory, it can be concluded that not all the measured bucket/single-pixel intensities are necessary for reconstruction, and that the bucket signal with larger values generally has a higher reconstruction contribution.

Now, let us review the definition of differential ghost imaging (DGI) [30], which greatly improves the quality of conventional GI. The bucket/single-pixel signal is defined as $S_{B}=\;\int \int \;I_{B}\left(x_{o},y_{o}\right)T\left(x_{o},y_{o}\right)dx_{o}dy_{o}$, where $I_{B}\left(x_{o},y_{o}\right)$ and $T(x_{o},y_{o})$ represents the intensity and transmission function at the spatial position $\left(x_{o},y_{o}\right)$ of the object *x* of p×q=N pixels, respectively. Similarly, the reference bucket signal (the total intensity of corresponding reference pattern) can be defined as $S_{R}=\;\int \int \;I_{R}\left(x,y\right)dxdy$, where $I_{R}\left(x,y\right)$ stands for the values at the spatial position (x,y) of reference speckle patterns. By using a differential bucket signal $S_{\Delta }=S_{B}-\frac{\langle S_{B}\rangle }{\langle S_{R}\rangle }S_{R}$ instead of $S_{B}$ in second-order correlation $\langle S_{B}I_{R}\left(x,y\right)\rangle$, where 〈⋯〉 signifies the ensemble average and $\frac{\langle S_{B}\rangle }{\langle S_{R}\rangle }$ is a constant, we can compute a differential ghost image ${\tilde{U}}_{\mathrm{DGI}}$ from M≫N measurements via ${\tilde{U}}_{\mathrm{DGI}}=\langle S_{\Delta }I_{R}\left(x,y\right)\rangle =\langle S_{B}I_{R}\left(x,y\right)\rangle -\frac{\langle S_{B}\rangle }{\langle S_{R}\rangle }\langle S_{R}I_{R}\left(x,y\right)\rangle$. A high image quality can be guaranteed by DGI, but at the cost of oversampling. Assume that the light field distribution of the source and the object beam are *O* and $O_{b}$, and that the minimum variation of the object transmission function *T* is denoted by $\Delta T_{\min}$, then we can get the signal-to-noise ratio (SNR) of recovered ghost image

(1) $${\mathrm{SNR}}_{\mathrm{GI}}=\frac{{\left[\Delta \langle O_{b}\rangle \right]}_{\min}^{2}}{\langle \Delta O^{2}\rangle }=\frac{M}{N_{\mathrm{speckle}}}\frac{\Delta T_{\min}^{2}}{\bar{T^{2}}},$$

where $N_{\mathrm{speckle}}=A_{\mathrm{beam}}/A_{\mathrm{coh}}$ indicates the number of speckles in the area of the light beam $A_{\mathrm{beam}}$. $A_{\mathrm{coh}}$ equals the square of each speckle size $\delta_{0}$, which can be determined by calculating the autocorrelation half height width of each point in the light field. For a fixed object, the term $\Delta T_{\min}^{2}/\bar{T^{2}}$ is constant, then SNR scales as $\frac{M}{N_{\mathrm{speckle}}}$. It means that making the speckle coherence area in each group change from large to small (i.e., making Nspeckle in each group change from small to large) can achieve better performance. Let us recall the RD method, it first divides the Hadamard basis sequence into four quarters and splits the first quarters in the next layer, and finally reorders the patterns within each quarter, also following the above rule, i.e., making the speckle coherence area for each pattern in every quarter change from large to small. Although this sorting method can produce a good image quality relative to that of CS, all quadrants (quarters) in each layer except the first quadrant are rough and contain too many patterns, which will cause many patterns in every quadrant of equal speckle coherence areas (i.e., resulting in multiple feasible solutions).

It is easy to understand that the random patterns are obviously not the best choices to minimize the number of measurements, because there is a high probability that half of all pixels transmit or reflect the light, which is more likely to generate an averaged single-pixel value, undoubtedly degrading image quality or weakening imaging efficiency. Moreover, the random sampling can be regarded as a relatively blind process with a large amount of redundant measurements. And it is really difficult for us to determine which random pattern has a higher reconstruction contribution or produces a higher bucket intensity. Although there are some definite matrices such as Toeplitz matrix [31] and polynomial matrix [32], which have been used in SPI and are much easier to be implemented, but their reconstruction qualities are generally much poorer than that of random measurement matrix. Recently, it was found that by using the two-dimensional polynomial to characterize optical aberrations at the pupil plane helps to recover more details of the object images and to decrease the average error [33].

According to common sense, the patterns that are highly similar or correlated with the object have a higher reconstruction contribution, i.e., there are many effective modulation spots falling within the object area. This undoubtedly requires us to know the object contour beforehand and let the shape of the patterns change with the target object, which is the main idea of adaptive compressive ghost imaging [12], i.e., adaptively sample the significant object regions at multi-scales. However, if the object is unknown or there is no rough profile measurements in advance, how can we generate a pattern that produces a higher measured value or reconstruction contribution? Fortunately, we find that, if we encode on the spatial light modulator (SLM) with a pattern consisting of all-one, which can be treated as a connected domain (CD, or block, which will be elaborated below), the detected intensity value will be the highest. If the pattern is divided equally into two CDs with the white-black pixels 50%:50%, then the detected value will be the second highest with a high probability. On this basis, a good construction of CDs in patterns can make a positive contribution to the image quality. To some extent, the area of CD is equivalent to the speckle coherence area. Therefore, the patterns with smaller number of CDs generally have the larger contributions to the recovery. Following above reasons and ideas, here we propose a novel origami pattern construction method, which makes full use of symmetric reverse folding (reverse the values on the corresponding pixels at the symmetric positions) and the axial symmetry of the rescaled pattern. The steps of our origami pattern construction are as follows.

**Step 1:** Assume that the pixel-sizes of patterns are all p×q=n square matrices, with the same size as the object, where *p*, *q* (p=q) are all even numbers. Suppose there are *n* patterns in total, we divide them into n/4 groups, each of which with four patterns. The group number is denoted by i=1,2,3,⋯,n/4. Note, as an initial setting, the first pattern $P_{1,1}$ in the first group is a matrix with all ones. Then, the second and the third patterns in this group $P_{1,2}$, $P_{1,3}$ are obtained by inversely folding the first one $P_{1,1}$ in this group up and down, left and right, respectively. That is, keeping the upper (or the left) half unchanged while the lower (or right) half is axisymmetric with the upper (or left) half but with its values in the lower (or right) half taking the opposite. By this means, we realize black (−1) and white (+1) inverting. The fourth pattern $P_{1,4}$ in this group is formed by performing both up-down and left-right reverse folding operations (the sequence of operations is interchangeable) on the first pattern $P_{1,1}$. Similarly, for all the subsequent groups, the last three patterns in the *i*th group, $P_{i,2}$, $P_{i,3}$ and $P_{i,4}$ will be generated from $P_{i,1}$ following the same processes:

(2) $$\begin{array}{cc} P_{i,2} & =\left[\begin{array}{c} P_{i,1}^{\mathrm{upper}}\\ -P_{i,1}^{\mathrm{lower}} \end{array}\right],P_{i,3}=\left[\begin{array}{cc} P_{i,1}^{\mathrm{left}} & -P_{i,1}^{\mathrm{right}} \end{array}\right], \end{array}$$

(3) $$\begin{array}{cc} P_{i,4} & =\left[\begin{array}{cc} P_{i,2}^{\mathrm{left}} & -P_{i,2}^{\mathrm{right}} \end{array}\right]=\left[\begin{array}{c} P_{i,3}^{\mathrm{upper}}\\ -P_{i,3}^{\mathrm{lower}} \end{array}\right]. \end{array}$$

**Step 2:** The first pattern $P_{i,1}$ in the *i*th group (also the 4(i−1)+1th pattern in the complete sequence) is built on the basis of the *i*th pattern in the current complete sequence:

(4) $$P_{i,1}=\left[\begin{array}{cc} P_{i\mathrm{th}}^{\mathrm{scaled}} & P_{i\mathrm{th}}^{\mathrm{scaled}}\\ -P_{i\mathrm{th}}^{\mathrm{scaled}} & -P_{i\mathrm{th}}^{\mathrm{scaled}} \end{array}\right].$$

**Figure 1:**
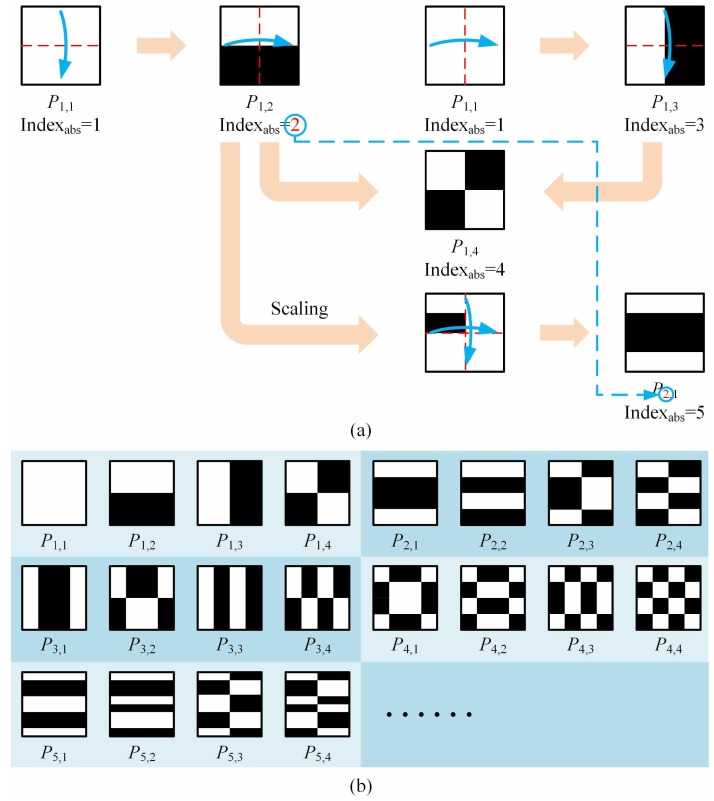
Origami pattern construction. (**a**) Pattern forming process for Steps 1–2. (**b**) Results obtained after Step 2.

Take $P_{2,1}$ in the second group for example; we firstly scale both horizontal and vertical pixel dimensions of the second pattern in the complete sequence (i.e., $P_{1,2}$) to their $\frac{1}{2}$ size, and place the scaled one on the upper left 1/4 part of a p×q square matrix with all zeros. Secondly, we perform the up and down, left and right axial symmetry about the midline lines on the vertical and horizontal axes of the matrix. After that, the pattern $P_{2,1}$ (also the 5th pattern in the complete sequence) is generated. Then, repeat Step 1 to acquire the second to fourth patterns in the second group, $P_{2,2}$, $P_{2,3}$ and $P_{2,4}$ (also the 6th to 8th patterns in complete sequence). By analogy, we can create all n/4 groups. Figure 1a gives a good illustration of Steps 1 and 2, and Figure 1b shows the result obtained after Step 2. To some extent, Step 2 is similar to the multiscale/pyramid methods, such as wavelet transforms. The front pattern with the absolute subscript *i* in the current complete sequence actually captures low-frequency characteristics (rough contours) of the target object, while the four patterns $P_{i,1}$$P_{i,2}$, $P_{i,3}$ and $P_{i,4}$ in the *i*th group are actually generated from their front pattern $P_{i\mathrm{th}}$ but with finer CDs and will undoubtedly add the details of high-frequency components with respect to the object. This also explains the operational mechanism of our method.

**Figure 2:**
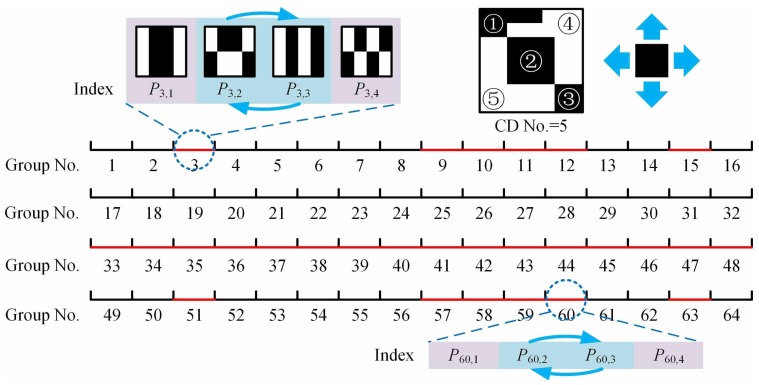
Group numbers for the cases of n=64, 256. The patterns $P_{i2}$ and $P_{i3}$ with red marked group numbers need to be order exchanged. The subgraph in the upper right corner illustrates an example of connected domains with a number of 5, but not related to the actual patterns used.

**Step 3:** Adjust the pattern sequence to ensure that the number of CDs for each pattern in the *i*th group is in an incremental order. Here, a CD is defined as an up-down-left-right connected area consisting of equal values (see the upper right corner of Figure 2, which is just an example to illustrate how a CD works, but not related to the actual patterns used). The neighborhoods on both sides of the axis may cause the partition of CDs, which is worth of investigating. Let us first look at the pattern $P_{i,1}$ whose pixel values on both sides of the symmetry axis are the same, its contribution to the recovery is the largest in the *i*th group. Since the pattern $P_{i,4}$ needs double symmetric reverse folding, its number of CDs is largest (with lowest reconstruction contribution) in the *i*th group. The patterns $P_{i,2}$ and $P_{i,3}$ are produced by only once symmetric reverse folding, whether to first perform up-down or left-right operation is approximately equivalent, thus they are particularly worth considering. As shown in Figure 2, the patterns $P_{i,2}$ and $P_{i,3}$ with a group indication (ID) (note: not the absolute subscript) i=3,9∼12,15 need to be order-reversed for the case of n=64, while those with a group ID i=3,9∼12,15,33∼48,51,57∼60,63 require adjustments for the case of n=256. This rule also exists in the higher-order cases. Given this, we set fourfold as a level. From top to bottom, the total n/4 groups (as a whole) can be catalogued into four parts: only the second part remains unchanged, the groups in the third part all need to be adjusted, while the groups in the first and fourth parts also need to be adjusted but with the same group IDs, depending on the recursive layer. Then, repeat the operation for the first part (as a new whole) until the number of groups in each new quarter part equals to 1. In the last layer, only the third part needs to be adjusted. Then, switch the order of the patterns $P_{i,2}$ and $P_{i,3}$ in these found groups.

**Figure 3:**
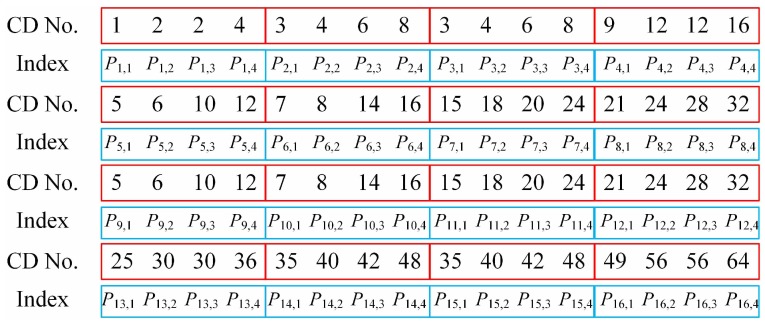
Numbers of CDs for a final pattern sequence of n=64.

**Figure 4:**
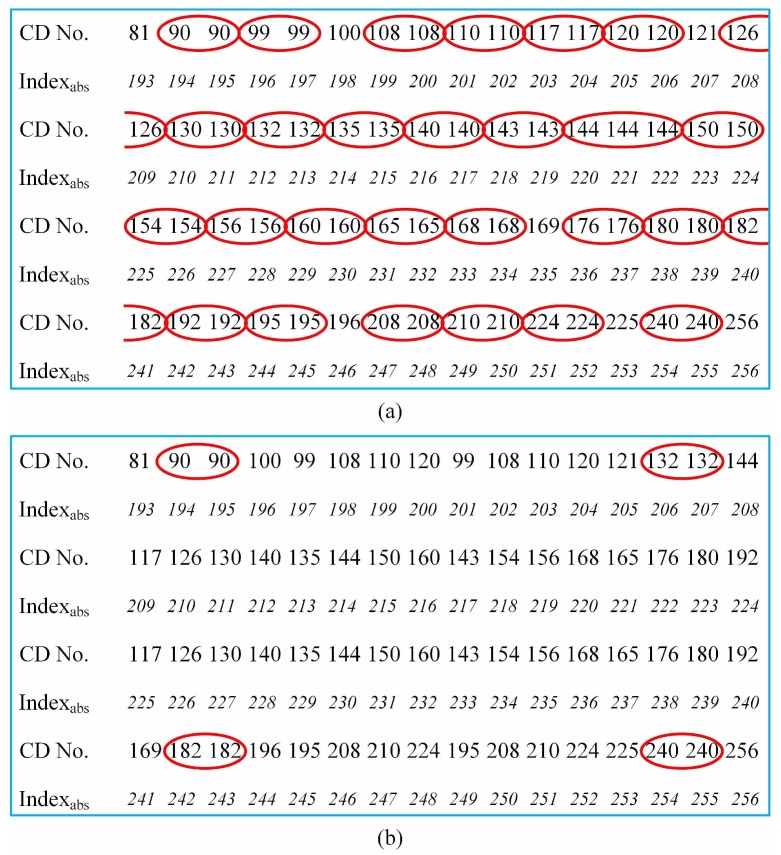
Comparison of the number of CDs between Russian dolls (RD) ordering of the Hadamard basis (**a**) and our method (**b**) in the high-order part. The patterns with the same CDs are circled by the red ellipses.

If we perform left-right and up-down reverse folding to generate $P_{i,2}$ and $P_{i,3}$ in Step 1, respectively, then the ID positions should be adjusted accordingly in Step 3. After the above three steps, we will get the final pattern sequence. Figure 3 gives an example of the final pattern sequence for n=64. In order to deeply analyze the advantages of our method, we compare it with the RD method. It is interesting to find that these two approaches have similar numbers of CDs in the low-order part (especially for the first 16 patterns, they are the same). Since their subsequent results are both derived from these first 16 patterns, it is advisable to set each 16 patterns as a comparison unit. Figure 4 shows the differences in their high-order parts (the last 64 patterns in 256-order sequence which also comprises the largest division of the RD method). As shown in Figure 4a, the RD ordering becomes very rough in this part, and has a lot of pattern pairs with the same number of CDs in each comparison unit. Because the maximum partition length of RD is n/4 and its minimum segmentation length is 4, it will inevitably incur too much uncertainty. Although the internal order exchange in these pattern pairs does not affect the RD sorting too much, it does bring hundreds of millions of possibilities of finding the optimal pattern sequence to get the best imaging quality, while in our scheme, each four patterns are treated as a group, i.e., the partition length is always 4. As shown in Figure 4b, there are only four red marks in our method for the high-order part, with only $2^{4}$ uncertainty. An intuitive explanation is that we rearrange the patterns into n/4 groups, each of which comprises four patterns, since the folding projections are implemented in the entire part or the first half part of one previous pattern with only four limited combinations. Fewer patterns with the same number of CDs in much smaller groups may make our method more accurate. Further mathematical interpretation will be the focus of our future work.

Now, each pattern $I_{R}$ can be sequentially reshaped into a row vector of 1×n, and then *n* such row vectors constitute a full-rank square matrix. It happens to be an orthogonal matrix and turns out to be a row-reordered Hadamard matrix in which only the row order is different from that of a natural-ordered Hadamard matrix. Such matrix is quite suitable for forming a measurement matrix $A\in R^{m\times n}$ of CS by just selecting the first *m* row vectors. Here, we use TVAL3 solver [34] to recover the image, in which our row number arrangement can be easily combined with its large-scale image reconstruction algorithm, and of course, our origami patterns are also available for GI.

## 3. Results

### 3.1. Numerical Simulations

Here, we introduce a unitless performance quantitative measure, the root mean square error (RMSE), which is defined as $\mathrm{RMSE}=\sqrt{\frac{1}{pq}\sum_{i,j=1}^{p,q}{\left[\tilde{U}\left(i,j\right)-U_{o}\left(i,j\right)\right]}^{2}}$. It describes the square root of differences between the recovered image $\tilde{U}$ and the original image $U_{o}$ for all pixels. Generally, the smaller the RMSE value, the better the quality of the recovered image.

**Figure 5:**
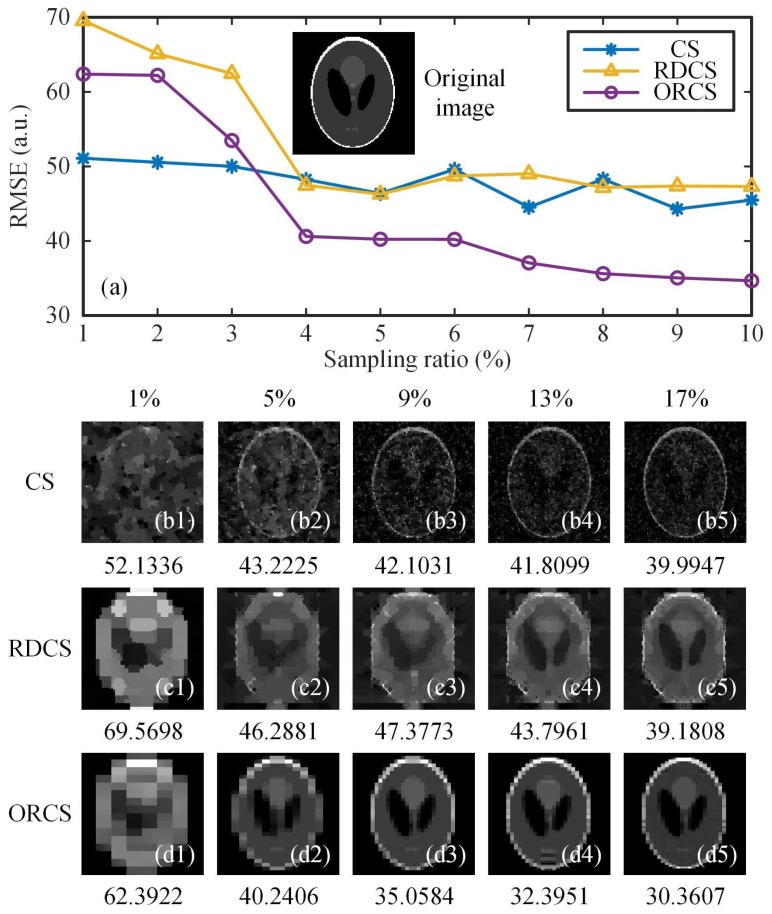
Reconstruction comparisons of the simple object, using CS, RDCS and ORCS. (**a**) gives an original head phantom image of 128×128 pixels and multiple RMSE curves as a function of the sampling ratio. (**b1**–**b5**), (**c1**–**c5**) and (**d1**–**d5**) are the CS, RDCS and ORCS reconstructions of 128×128 pixels at 1%–17% sampling ratios, respectively. The digits below (**b1**–**d5**) are the RMSE values.

To test whether the measurement data acquired by our sensing method can generate better reconstruction performance than other approaches, some numerical calculations were performed. All calculations were performed on an ordinary laptop with an Inter Core i7-8650U central processing unit (CPU) @ 1.90 GHz and a random access memory (RAM) of 16 GB. For a fair comparison, we also combined the RD ordering of the Hadamard basis with CS, instead of second-order correlation used in the original version [21], and we call it the Russian dolls compressed sensing (RDCS). In the first simulation, a head phantom image on a black background of 128×128 pixels was used as an original image, thus, the reconstructed images also had resolutions of 128×128 pixels. In Figure 5, for conventional CS, we used a totally random 0/1 binary measurement matrix, which is widely applied in SPI schemes because the digital micromirror device (DMD) can only be encoded with 0/1 patterns. For implementing positive–negative light intensity modulation with the matrices consisting of positive and negative binary numbers, one needs to make a differential measurement between two patterns, which requires twice the actual amount of measurements. That is, to align to a 100% sampling rate of 0/1 random matrices, we should double the 50% sampling rate of positive-negative matrices. All sampling rates below were calculated in this way. The RMSE curves of CS, RDCS and origami compressed sensing (ORCS) as a function of the sampling ratio (from 0.5% to 10%) were given in Figure 5a, and their retrieved images at different sampling ratios from 1 to 17% with a 4% stepping increase are presented in Figure 5(b1–d5). All these methods show a similar trend that the image quality is approximatively proportional to the sampling ratio. For the small sampling ratios, conventional CS and RDCS perform very similarly. When the sampling ratio is large enough, the RDCS will perform better than traditional CS. From the data, it can also be seen that the performance of our method outperforms those of CS and RDCS for the simple object. For this case, only 9% measurements are enough for ORCS to obtain a nice reconstruction. The reason why ORCS exhibits less noise than RDCS is that ORCS performs the reordering of the Hadamard matrix with only four patterns in each group, and its sort is much more refined than that of RDCS.

**Figure 6:**
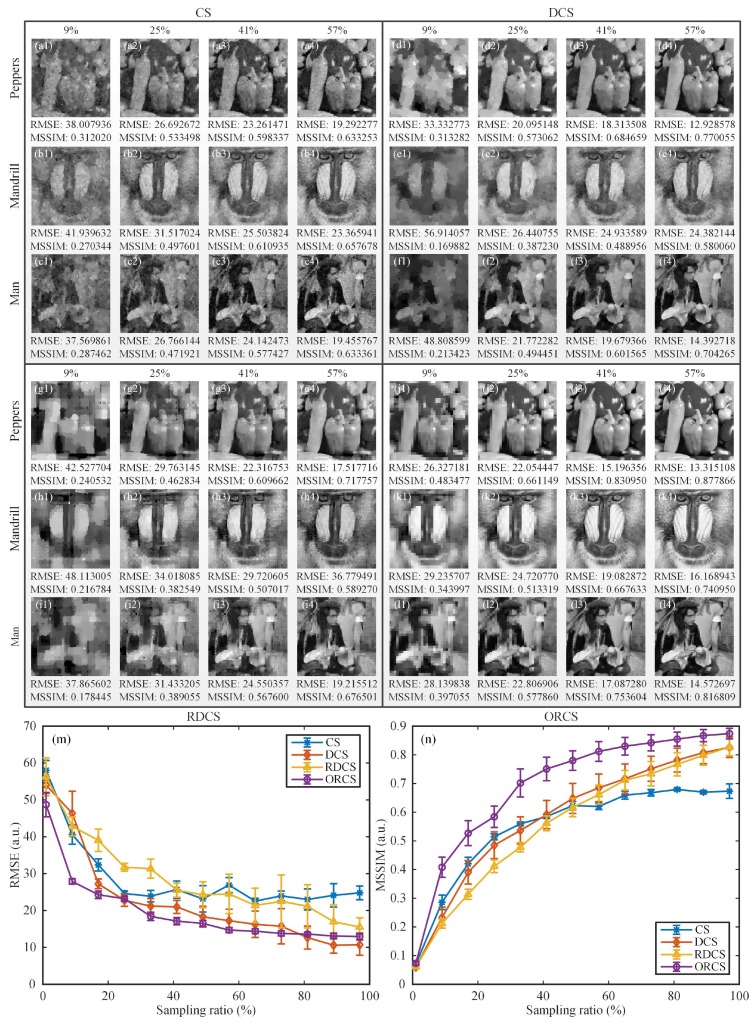
Reconstruction comparisons for general scenes. (**a1**–**c4**), (**d1**–**f4**), (**g1**–**i4**) and (**j4**–**l4**) are the recovered images of 128×128 pixels at the sampling ratios of 9%, 25%, 41% and 57%, using CS, DCS, RDCS and ORCS, respectively. Below each image, the RMSE and MSSIM values are also provided. (**m**) and (**n**) are the comparisons based on the RMSE and MSSIM value as a function of the sampling ratio, respectively. The sampling ratio ranges from 1 to 97% with an 8% stepping increase. Every data is acquired by averaging the RMSE or MSSIM values of three different object samples, along with a error bar whose height indicates the standard deviation of each point.

In the next simulation, we investigated the average imaging performance of our method for more general complex scenes. To exclude that the proposed method only works well for simple images that are similar to the head phantom, we chose three typical gray-scale test images, i.e., the pictures of peppers, mandrill and man. Figure 6 illustrates the comparison results of these objects. As we know, RMSE as a pixel-wise performance metric may fail to describe the visible structures or perceptual quality of natural images, and somtimes cannot correctly tell the image quality. Therefore, in addition to RMSE, another quantitative measure mean structural similarity (MSSIM) is also used to evaluate the image quality, based on the perceptual difference between a reference and a processed image. To some extent, the MSSIM value reveals not only the reconstruction error, but also the structural distortion of the recovered images. Its value ranges from 0 to 1; the larger the MSSIM value, the better the image quality. Since the traditional CS for SPI uses a binary measurement matrix consisting of 0 or 1, while the Hadamard matrix takes values of −1 or 1. For the sake of fairness, we added the differential CS (DCS) [35,36,37] to the comparison. The DCS applies a measurement matrix that follows positive-negative distribution, take the ±1 measurement matrix $I_{R}$ as an example, it can subtly shift and stretch $I_{R}$ to two complementary matrices ${\hat{I}}_{R}=\left(I_{R}+1\right)/2$ and ${\overset{ˇ}{I}}_{R}=1_{N\times N}-{\hat{I}}_{R}$, where $1_{N\times N}$ stands for an all 1 matrix, then followed by their difference to realize differential modulation $I_{R}={\hat{I}}_{R}-{\overset{ˇ}{I}}_{R}$.

The reconstruction comparisons of CS, DCS, RDCS and ORCS for more general scenes at the sampling ratios of 9%, 25%, 41% and 57% are presented in Figure 6(a1–c4,d1–f4,g1–i4,j4–l4), with the RMSE and MSSIM values being indicated below each image. In terms of images and performance evaluation parameters, we can clearly see that the image quality of ORCS is much better than those of other three approaches under the same sampling ratios. We further drew their RMSE and MSSIM curves of recovered images as a function of the sampling ratio, as shown in Figure 6m–n. Although the scene has more spatial complexity, the performance trends of these methods keep in accordance with the case of simple object. From Figure 6n, we can see that the performance of ORCS is generally better than the other three methods with an overwhelming superiority for all sampling ratios, while the reconstruction quality of RDCS and DCS is very close, which is coincident with the theory of RD [21]. In other words, to acquire the same reconstruction quality, our ORCS requires a sampling ratio that is much closer to the upper bound specified by the phase transition of CS reconstruction. In addition, when the sampling rate is below 49%, CS, DCS and RDCS behave similarly, while for the sampling ratios above 49%, both RDCS and DCS outperform the traditional CS. This is because both the RDCS and DCS actually realize positive–negative intensity modulation, and the RD reordering of the Hadamard matrix is too rough, thus, the RDCS cannot show obvious advantages over the DCS. When the sampling ratio is large enough, the RMSE and MSSIM values of all approaches tend to saturate.

**Figure 7:**
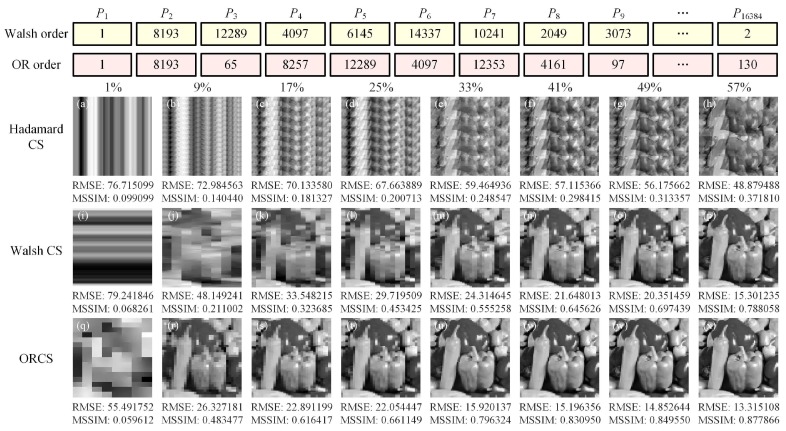
Comparisons of reconstruction results using natural-ordered (**a**–**h**), Walsh-ordered (**i**–**p**) and origami-ordered (**q**–**x**) Hadamard patterns. For comparison, the pattern number of each Walsh–Hadamard pattern and origami pattern with respect to the original sequence of the natural-ordered Hadamard patterns is given at the top.

The proposed method provides an alternative row-reordering of the Hadamard matrix. To compare with the classic Walsh–Hadamard patterns, we gave the pattern number of each Walsh–Hadamard pattern and origami pattern with respect to the original sequence of natural-ordered Hadamard patterns, as shown in Figure 7. As we know, the Walsh-ordered Hadamard matrix also performs row-reordering of natural-ordered Hadamard matrix, by rearranging the rows based on an increasing order of the number of ±1 sign changes of each row via the bit-reversal permutation followed by the Gray-code permutation. Therefore, whether in principle or in the reordering sequences, the origami sort and the Walsh sort are different. Next, we presented the image reconstructions using natural-ordered, Walsh–Hadamard patterns and origami patterns, respectively, as shown in Figure 7a–x. It is obviously seen from the recovered images that when we perform the subsampling, their reconstruction results are definitely different from each other. When using the natural-ordered Hadamard patterns, there exists some periodic silhouette ghosting in the recovered images, caused by the periodic structure of the matrix itself. When using the Walsh–Hadamard patterns, the reconstructed images at low sampling ratios have horizontal stripes. Our origami patterns can avoid these negative effects and yield relatively better performance. Now, let us delve into the essence of our method. As we know, the Hadamard patterns generally have various spatial frequencies. In each group of the origami patterns, the front patterns have a smaller number of CDs (i.e., larger blocks), forming the low-frequency components (rough contour) of the object, and the following patterns have a larger number of CDs (i.e., smaller blocks), contributing to the high-frequency components (details) of the target. Since the first origami pattern in the *i*th group is derived from the *i*th origami pattern in the complete sequence, the mean of the number of CDs in each group is also increasing with the group number. Therefore, patterns with a smaller group number capture low-frequency features of the object image, while those with larger group number will supplement the details of high-frequency components. This explains how our method works and why it outperforms the others.

**Figure 8:**
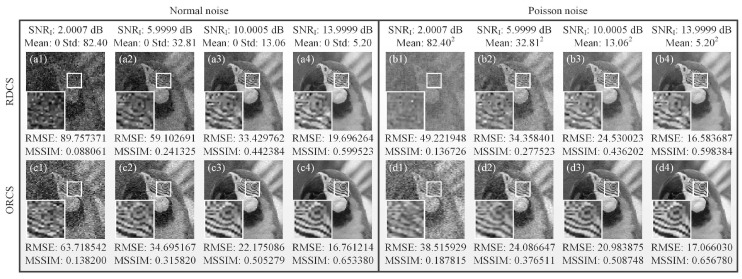
Reconstructed images of both RDCS and ORCS of 128×128 pixels, all with a 90% sampling ratio, under different illumination fluctuation noise. (**a1**–**a4**) and (**b1**–**b4**) illustrate the reconstructions of RDCS under the noise that follows the normal and Poisson distribution, respectively. (**c1**–**c4**) and (**d1**–**d4**) separately present the recovered images of ORCS under the normal and Poisson noise.

Next, we simulated the influence of illumination fluctuation noise on image reconstructions. As we know, in mathematical models, all noise in SPI system can be regarded as the additive noise, thus, the effects of different noise sources involved in the imaging process are similar. Here, we directly added the illumination fluctuation noise [38] on the target to be detected. The SNR of the spatial light field distribution I(j) of illumination fluctuation noise can be calculated by ${\mathrm{SNR}}_{I}=10\log_{10}\frac{\langle I(j)\rangle }{\mathrm{Std}(\mathrm{noise})}$, where Std signifies the standard deviation. Two kinds of noise which follow the normal and Poisson distributions are set for both RDCS and ORCS methods. In Figure 8, compared with RDCS, the details of reconstructed images using ORCS is much easier to identify from the enlarged part of the white square frame in the lower left corner of each image, under the same ${\mathrm{SNR}}_{I}$ for either of the above two types of noise, and the image quality gradually improves with the increase of the ${\mathrm{SNR}}_{I}$ value.

### 3.2. Experimental Setup and Results

**Figure 9:**
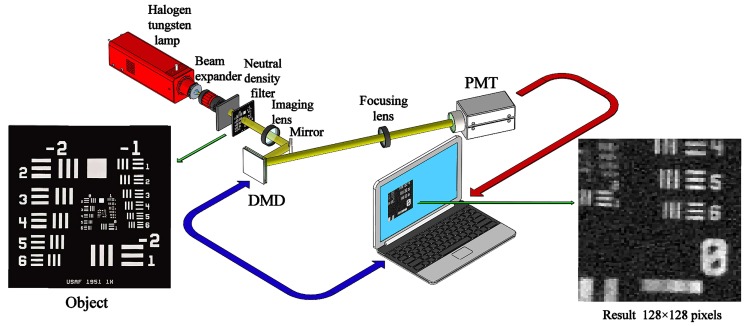
Schematic of the experimental setup. After being collimated and broadened, the light illuminated the object (a negative 1951 USAF resolution test chart of 3 inch × 3 inch) and then it was imaged on the DMD. A PMT collected the differential photon counts via a focusing lens. The ORCS reconstructed image of 128×128 pixel-size at 60% sampling ratio is given in the lower right corner.

**Figure 10:**
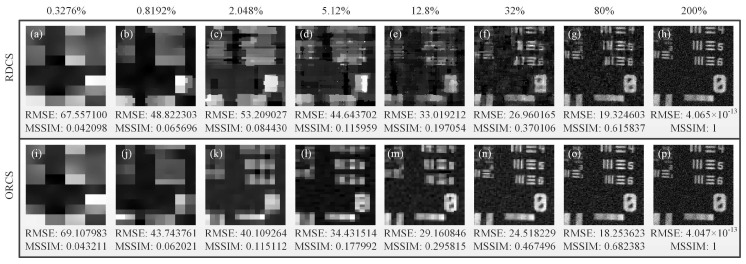
Experimental results of RDCS and ORCS under varying sampling ratios changing from 0.3276% to 200% with a 2.5× stepping increase. The RMSE and MSSIM values are also given, treating the reconstructed image with full sampling as a reference image. The image quality progressively improves with the increasing number of measurements.

Our experimental realization was based on a conventional single-pixel camera, as shown in Figure 9. The thermal light from a stabilized halogen tungsten lamp with a wavelength of 360∼2600 nm was collimated and attenuated via a beam expander and a neutral density filter (NDF), respectively. Here, the NDF was used to attenuate the light to the ultra-weak light level. The attenuated light passed through a negative 1951 USAF resolution test chart, and then was imaged onto the DMD (one SLM) via an imaging lens and a mirror. The applied DMD consists of 1024×768 micromirrors, each of which can be switched between two directions of $\pm 12^{{}^{\circ}}$, corresponding to 1 and 0. Since the values of our patterns are either 1 or −1, we needed to divide each generated ±1 pattern $I_{R}$ into a complementary matrix pair [35,36,37] ${\hat{I}}_{R}$ and ${\overset{ˇ}{I}}_{R}$, which was sequentially encoded on the DMD. A counter-type Hamamatsu H10682-210 PMT was placed on one of the reflection orientations to accordingly make a differential measurement of two adjacent total photon counts. By using the ORCS method, we acquired a very satisfactory image quality, as shown in the right illustration of Figure 9. For comparison, we also gave the experimental results of RDCS and ORCS at different sampling ratios, as presented in Figure 10. It can be easily seen that the image quality is proportional to the number of measurements, and our ORCS outperforms RDCS, which is consistent with the simulation results. When we perform full sampling, the performance of RDCS and ORCS will be almost the same, because their measurement matrices in this case only have inconsistencies in the row order. In our experiment, the sampling frequency of the PMT is set to 500 Hz. For the object of 128×128 pixel-size with a total sampling ratio of 32%, it only takes 10.49 s for both RDCS and ORCS to finish data acquisition and takes 31.90 s (averaged calculation time) for image reconstruction running on an ordinary laptop, i.e., the total imaging time is 42.39 s. It is worth mentioning that the maximal binary pattern switching rate of the DMD is 32550 Hz, if we set the working frequency of the PMT to 32550 Hz and use a computing device with a better configuration, the total imaging time can be further reduced.

## 4. Conclusions

In conclusion, a single-pixel compressive imaging method with origami pattern construction is proposed, where the patterns with the biggest contributions to the recovery are preferentially displayed. By symmetric reverse folding, axial symmetry and partial pattern order adjustment, the generated deterministic patterns ensure better image quality but with less uncertainty of the pattern sequence, compared with traditional CS, DCS and RDCS. The ORCS approach has been demonstrated experimentally with a classic SPI setup using differential modulation technique. Both simulation and experimental results prove that the sampling ratio of the proposed method can be reduced, much closer to the upper bound specified by the phase transition of CS reconstruction, with a potential of promoting single-pixel compressive video applications.
